# CoreSNP: an efficient pipeline for core marker profile selection from genome-wide SNP datasets in crops

**DOI:** 10.1186/s12870-023-04609-w

**Published:** 2023-11-21

**Authors:** Tingyu Dou, Chunchao Wang, Yanling Ma, Zhaoyan Chen, Jing Zhang, Ganggang Guo

**Affiliations:** Key Laboratory of Grain Crop Genetic Resources Evaluation and Utilization (MARA), The National Key Facility for Crop Gene Resources and Genetic Improvement, Institute of Crop Sciences, Chinese Academy of Agricultural Sciences (ICS-CAAS), Beijing, 100081 China

**Keywords:** Shannon index, Germplasm discrimination and management, Low-dense genotyping

## Abstract

**Background:**

DNA marker profiles play a crucial role in the identification and registration of germplasm, as well as in the distinctness, uniformity, and stability (DUS) testing of new plant variety protection. However, selecting minimal marker sets from large-scale SNP dataset can be challenging to distinguish a maximum number of samples. Results: Here, we developed the CoreSNP pipeline using a “divide and conquer” strategy and a “greedy” algorithm. The pipeline offers adjustable parameters to guarantee the distinction of each sample pair with at least two markers. Additionally, it allows datasets with missing loci as input. The pipeline was tested in barley, soybean, wheat, rice and maize. A few dozen of core SNPs were efficiently selected in different crops with SNP array, GBS, and WGS dataset, which can differentiate thousands of individual samples. The core SNPs were distributed across all chromosomes, exhibiting lower pairwise linkage disequilibrium (LD) and higher polymorphism information content (PIC) and minor allele frequencies (MAF). It was shown that both the genetic diversity of the population and the characteristics of the original dataset can significantly influence the number of core markers. In addition, the core SNPs capture a certain level of the original population structure.

**Conclusions:**

CoreSNP is an efficiency way of core marker sets selection based on Genome-wide SNP datasets of crops. Combined with low-density SNP chip or genotyping technologies, it can be a cost-effective way to simplify and expedite the evaluation of genetic resources and differentiate different crop varieties. This tool is expected to have great application prospects in the rapid comparison of germplasm and intellectual property protection of new varieties.

**Supplementary Information:**

The online version contains supplementary material available at 10.1186/s12870-023-04609-w.

## Background

Accurate identification and registration of germplasm resources are essential for plant conservation efforts [1–3]. Traditional identification methods based on morphological or agronomical traits can be time-consuming due to the influence of environmental factors on most phenotypes [4]. Genetic molecular markers have been recommended by the International Union for the Protection of New Varieties of Plants (UPOV) as a more reliable and efficient approach for variety and cultivar identification, in addition to morphological characteristics [5–7].

With the increasing number of germplasm collections stored in genebanks, effective characterization and management have become major challenges [8]. In the USDA Soybean Germplasm Collection, it was discovered that over 30% of wild accessions and 23% of cultivated accessions were redundant, as their similarity exceeded 99.9% [9]. Similarly, an analysis of genetic profiles in the German ex situ gene bank revealed that approximately 33% of the 22,626 collections of barley (*Hordeum vulgare* L.) were potential duplicates [10]. By selecting a comprehensive core set of molecular markers, germplasm collections can be rapidly assessed and characterized. Assigning a unique “molecular passport” profile to each accession enables researchers to easily track and manage the collections, reducing duplication and facilitating targeted utilization [11].

Furthermore, core marker sets play a vital role in protecting new plant varieties. Due to the increasing number of crop varieties being released onto the market, the limited genetic diversity and close similarity among elite parental lines lead to fewer morphological differences that can be utilized for variety identification in modern breeding programs [12]. The preselected small set of markers can be cost-effectively utilized for DUS testing, identification of essential derived varieties (EDVs) and the verification of seed authenticity and purity. This benefits breeders by protecting their intellectual property rights [13–15].

Among various types of molecular markers, single nucleotide polymorphisms (SNPs) have gained significant importance due to their high reproducibility, locus specificity, and wide distribution throughout the genome [16]. The continuous advancements in high-throughput sequencing technology have facilitated the generation of a large number of SNPs. Through various genotyping platforms, researchers can obtain millions of SNPs that cover the entire genome, resulting in SNP sets with diverse characteristics [17–19]. The reduction of SNP density and the development of low-density SNP genotyping panels have gained prominence due to its cost-effectiveness, enabling the identification of large-scale germplasm and the assessment of their relatedness. A variety of genotyping methods and technologies, such as Taqman genotyping assays [20], Kompetitive allele-specific PCR (KASP) [21, 22], Amplification refractory mutation system PCR (ARMS-PCR) [23], as well as low density SNP chips [24] have been extensively used in genotyping specific SNPs of interest. Selecting the fewest and most representative SNPs from vast amounts of information that contain redundant data has become a concern for researchers.Currently, various methods for selecting core SNPs selection have been adopted in various crop species for variety identification and DNA fingerprinting [25–28]. In soybean, Liu et al. divided 4044 SNPs into 24 panels with varying numbers of SNPs based on polymorphic information content (PIC) values. A core set panel of 20 SNPs was selected to construct molecular IDs for 138 released soybean cultivars, resulting in the fewest number of indistinguishable pairs of accessions [29]. Using a combination of polymorphisms and principal component analysis, Li et al. selected 60 core SNPs distributed across all chromosomes to provide sufficient genetic information for 166 representative inbred lines of Chinese cabbage from a pool of 1167 SNPs [30]. However, these studies did not consider the discriminatory power of combinations of SNPs, and the manual screening process used in these studies may not be sufficient to meet the increasing demand for selecting core SNPs from large-scale sequenced data.

Automated methods based on computer programming have also been proposed to improve the efficiency of selecting core SNPs. These methods aim to construct relatively small marker sets capable of distinguishing a wide range of varieties. Hiroshi et al. (2013) employed an exhaustive method to develop MinimalMarker software for identifying minimal marker sets. They constructed a pairwise comparison matrix by calculating the number of differential alleles between each pair of varieties and consistently selected the marker with the highest discrimination to form the minimal set [31]. This algorithm was subsequently used for core SNP selection in the identification of pepper [32] and cucumber varieties [33]. In their study on rice, Yuan et al. introduced a method called conditional random selection (CRS) to specifically distinguish between EDV and non-EDV varieties [34]. The method follows a “divide and conquer” strategy, where specific haplotypes are initially constructed using randomly selected SNPs. Redundant SNPs were then eliminated by systematically shielding one SNP at a time while checking whether the remaining SNPs could still distinguish all varieties. Through this approach, the researchers selected a set of 390 SNPs that could distinguish between 3,024 rice varieties.

Despite the progress made in selecting core SNPs, these studies are relatively time-consuming and not user-friendly, especially when dealing with data that contain missing loci. Here, we propose CoreSNP, a novel and customized pipeline for developing minimal core SNPs. This pipeline can largely reduce the number of SNPs essential for identifying different varieties from large-scale genotyping data. Based on publicly available genotype datasets, the pipeline has been proven to be robust in different crops and across various sequencing platforms.

## Materials and methods

### Description of the coreSNP pipeline

**Fig. 1:**
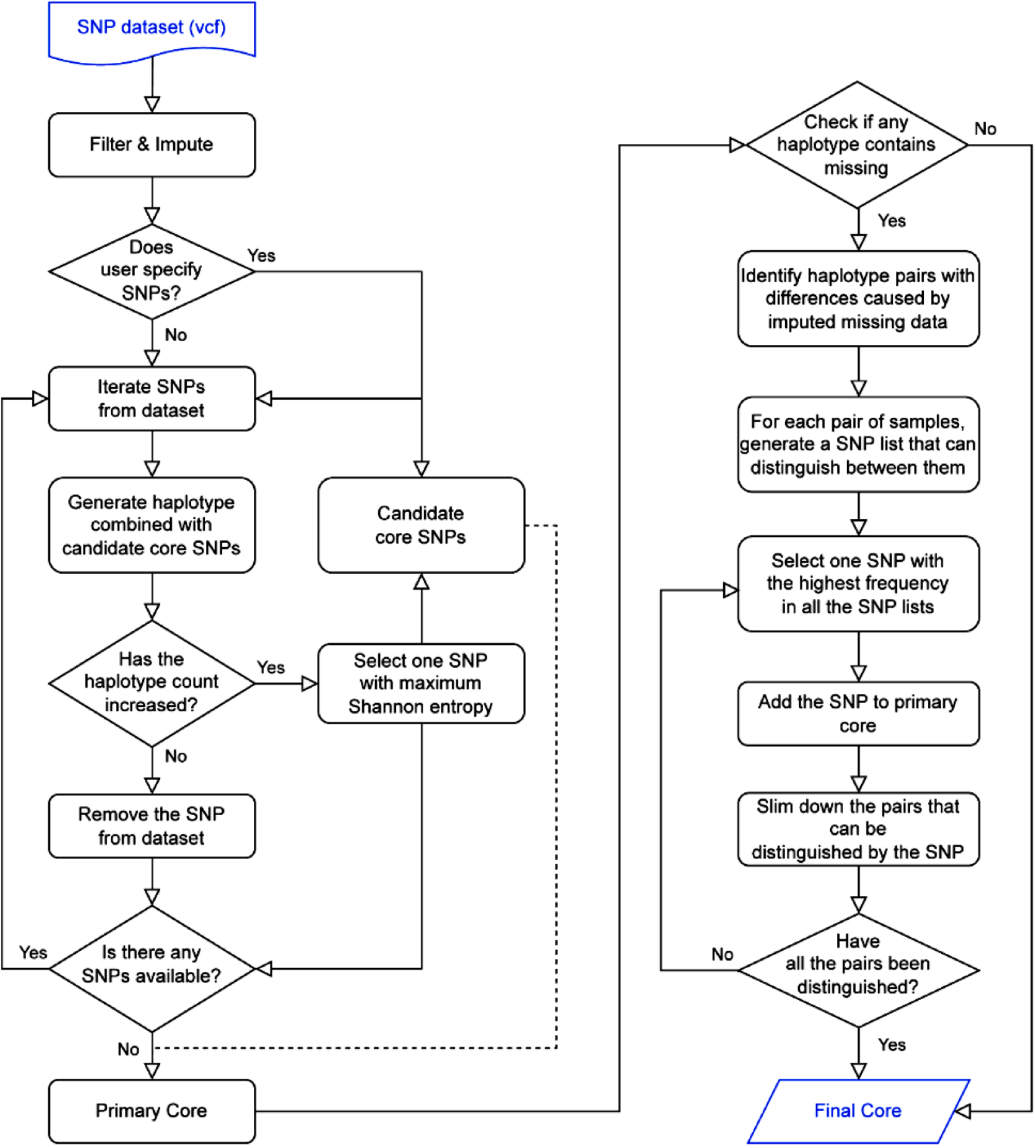
The basic workflow of the CoreSNP pipeline

**Fig. 2:**
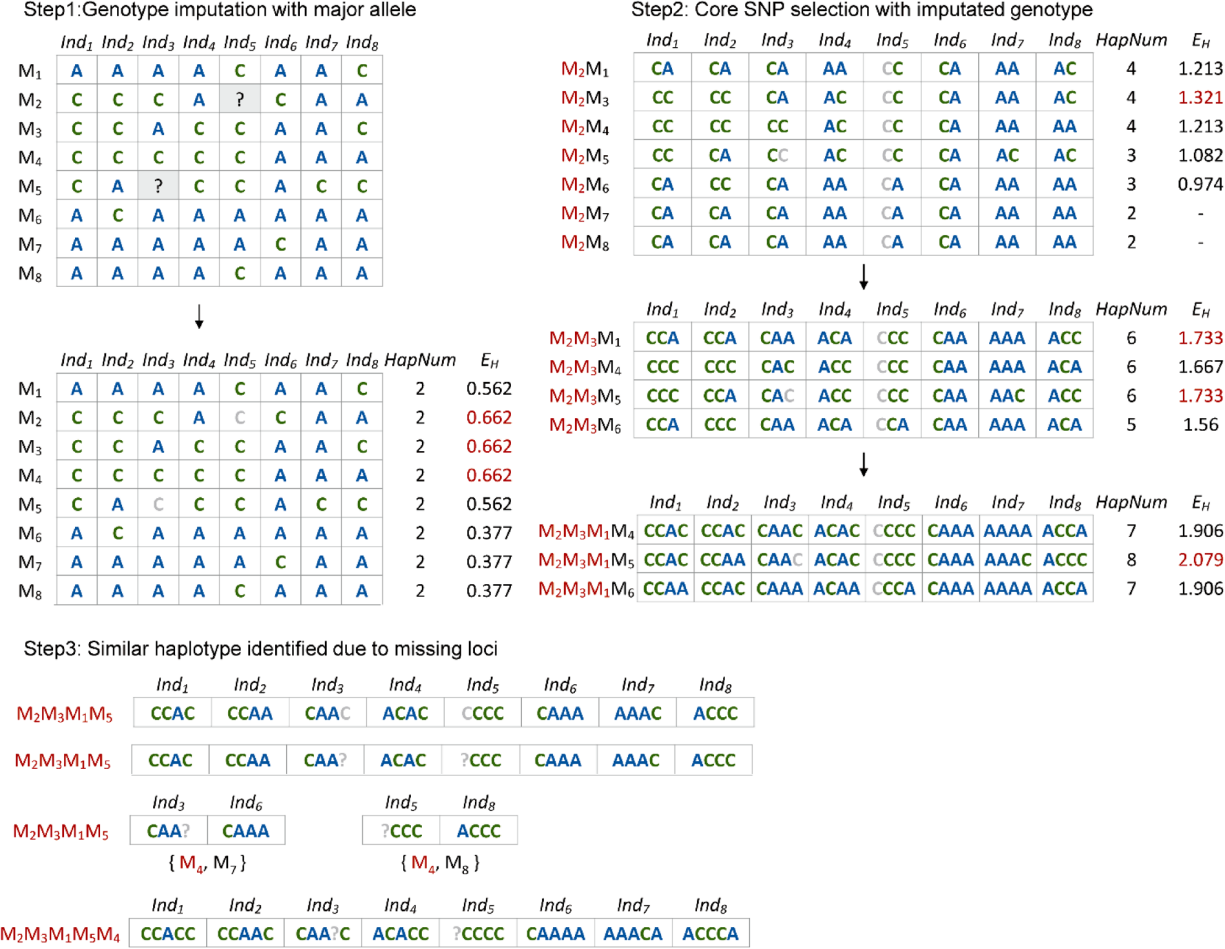
Schematic diagram of the CoreSNP pipeline

A greedy search algorithm was applied to the core SNP selection and the specific workflow of the pipeline is summarized in Fig. 1. To provide a clear illustration of the selection process, we took the dataset with 8 individuals and 8 markers as an example with a schematic diagram (Fig. 2). In the initial step, missing SNPs were imputed by major homozygous SNP genotype. The Shannon index was calculated for each SNP was subsequently calculated for each SNP in the dataset, and the SNP with the highest value was randomly selected as the first solution. The pipeline then proceeded by iteratively analyzing the remaining SNPs in the dataset and constructing the haplotypes using the selected SNPs. Next, the SNP that maximized the Shannon entropy index was selected as the second solution and accordingly removed from the dataset correspondingly. The iterative process continues until no additional SNPs can be added. During this procedure, a crucial step involvesassessing whether the newly selected SNP contributes to an increase in the number of observed haplotypes before calculating the Shannon index. If not, the SNP is skipped, thereby expediting the pipeline. As depicted in Fig. 2, SNPs such as ‘*Marker*7’ and ‘*Marker*8’ were used as examples. In this step, we exclude SNPs without polymorphisms and nearby SNPs linked to the selected core SNPs. Consequently, at each selection step, the process achieved the local optimal solution to maximize sample differentiation and haplotype diversity.

After employing major allele imputation, the haplotypes without any missing values were outputted, and the chosen SNPs were designated as the primary core markers. Recognizing that missing sites could potentially result from sequencing errors, we retrieved haplotypes with missing loci based on the positions of imputed values. Subsequently, the pipeline identified pairs of haplotypes with differences caused by imputed missing data. Through an iterative process, SNPs with the highest frequency were selected and added to the primary list. Eventually, the final core sets were generated after achieving complete differentiation across all sample pairs (Fig. 2).

In this study, additional command options were introduced to configure the running parameters, thereby enhancing the applicability of the pipeline. Users can define input files using the “-v” option and customize which markers should be included or excluded using the “-i” and “-e” options, respectively. Since some SNP combinations (haplotypes) have close Shannon index values, the “-x” option was provided to allow users to specify the number of candidate SNP combinations to be selected in each round. This option provides users with more feasible solutions for downstream analysis. Furthermore, core SNPs were randomly and independently selected each round, and users have the flexibility to define the number of repetitions using the “-c” option. The “-m” option allows users to specify that at least two markers are required to effectively discriminate between every pair of varieties. This criterion can indeed mitigate potential errors or noise introduced by the raw sequencing data, thereby enhancing the reliability and robustness of the results (Table S1).

### Genotype dataset collection

To validate the CoreSNP pipeline, we collected the public SNP genotype dataset of barley, soybean, wheat, rice, and maize from widely studied plant databases or research studies (Table S2). The SNP data were obtained from different genotyping and sequencing platforms, including SNP microarray, high-throughput genotyping by sequencing (GBS), and whole-genome sequencing (WGS).

For the barley analysis, genotypic information of 1,000 accessions was downloaded from the Germinate Barley SNP Platforms [35]. The collection of 1,000 genotypes is a representative subset of the global 22,626 barley accessions from the German Federal ex situ GenBank. By integrating our unpublished data, we curated a dataset consisting of 1081 barley accessions with 42,520 SNPs genotyped by the 50 K iSelect SNP Array, referred to as Barley**I**. Additionally, we obtained a GBS SNP matrix of 1,297 barley accessions from the IPK data repository, referred to as Barley**II** [10]. We merged two different datasets by using a shared sample set of 1081 barley accessions. The resulting combined dataset, designated Barley **I **&** II**, contains a total of 185,508 SNPs.

The soybean dataset comprises 817 soybean accessions with 158,959 SNPs, including 77 parental lines, 169 nonparental lines, and 571 progenies. Genotyping was performed using the ZDX1 array genotyping platform [36]. The wheat genotypic dataset consists of 178,803 SNPs and includes genotypic information for 271 Chinese wheat landraces that were genotyped using the 660 K wheat SNP array [37].

We extracted genotypic information from 453 high-coverage rice accessions obtained from the 3,000 Rice Genome Project [38]. The maize genotypic dataset includes genotype information from 1,210 maize lines based on whole-genome sequencing data, comprising approximately 83 million raw SNPs. The data were downloaded from the maize HapMapV3 study [39].

### Data preprocessing

Heterozygous sites were treated as missing data and processed using BCFtools version 1.10.2 [40]. Genotype imputation of missing sites was performed using FILLIN with default parameters [41]. Sample and SNP filtration as well as LD-based SNP pruning were performed using PLINK version 1.90 [42].

### Polymorphism analysis

In this study, the Shannon indices [43] and PIC values [44] were calculated using the following formulas.

1 $$H = - \sum _{i = 1}^n{P_i}ln\left( {{P_i}} \right)$$

where *H* is the diversity index, *n* is the population size and *P*_*i*_ is the frequency of the combined haplotypes.

2 $$PIC = 1 - \sum _{i = 1}^n{P_i}^2$$

3 $$PIC = 1 - \sum _{i = 1}^n{P_i}^2 - \sum _{i = 1}^{n - 1}\sum _{j = i + 1}^n2{P_i}^2{P_j}^2$$

where n is the population size and *P*_*i*_ and *P*_*j*_ are the frequencies of two SNP alleles among all samples. Equation 2 suggests the simplified formula, while Eq. 3 suggests the full formula for PIC calculation.

### Genetic diversity analysis

Allele counts and frequencies and IBS information were calculated by PLINK version 1.90 [42]. LD estimation was measured as parameter r^2^ with a maximum distance of 10 Mb using PopLDdecay version 3.40 [45]. A Mantel test for correlation between distance matrices was performed using an in-house python script [46]. Principal component analysis (PCA) of the barley samples was performed using EIGENSOFT/smartPCA software version 6.1.4 [47]. All figures were generated using R software version 4.1.2 using the packages ggplot [48] and RIdeogram [49].

### Data availability

The CoreSNP pipeline was developed using the Python programming language and accepts compressed Variant Call Format (VCF) files or uncompressed VCF files as input. The cost-free program is readily executed from the command line, relying on specific dependencies, such as Python (version 3.6 or higher), Numpy and PLINK 1.9. Both the source code and the essential dependencies are accessible on GitHub (https://github.com/admy55/CoreSNP) or Gitee (https://gitee.com/admy55/CoreSNP).

## Results

### Performance test of the CoreSNP pipeline

**Fig. 3:**
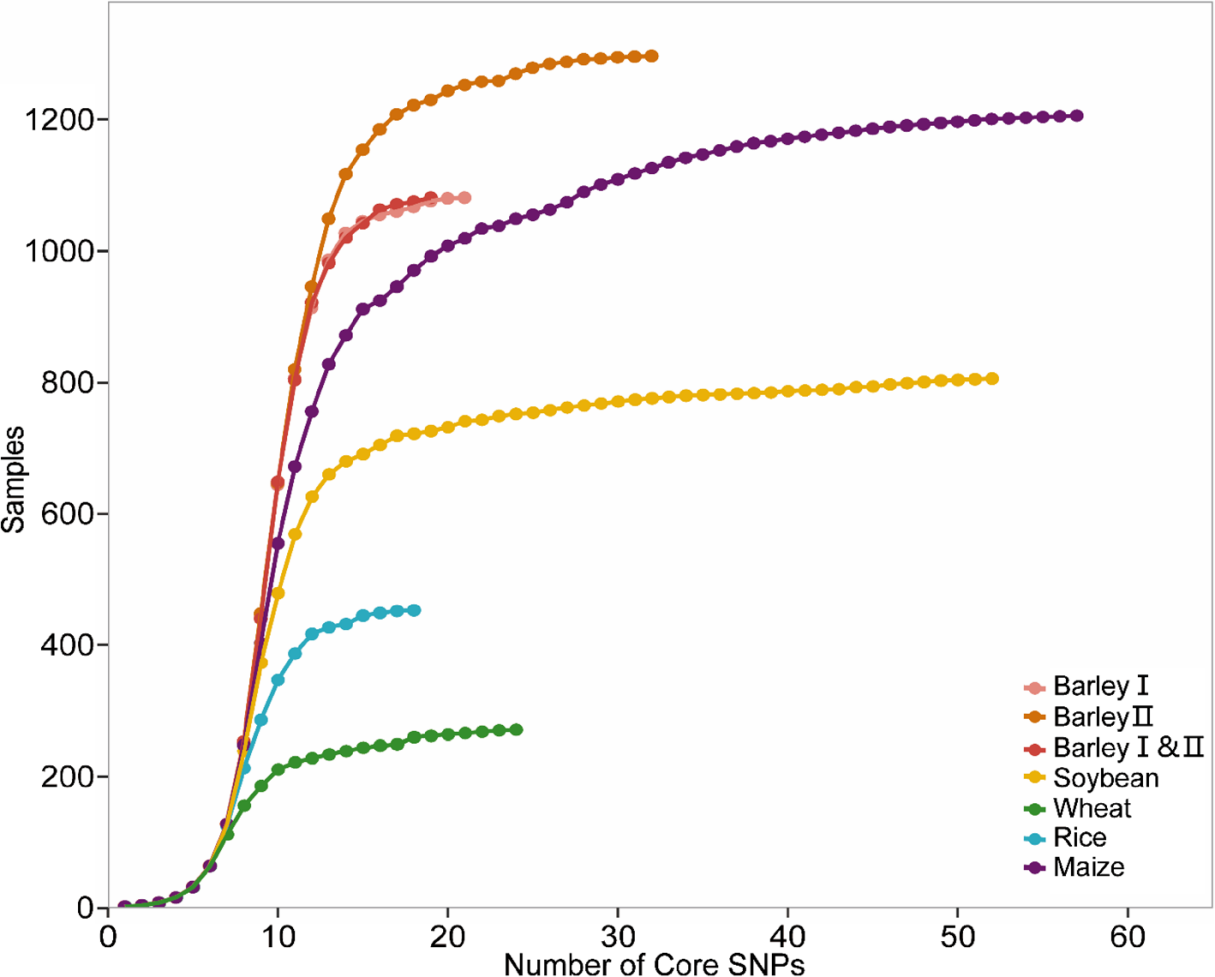
Discrimination saturation curve of core SNPs selected from raw dataset

Initially, imputation and filtration were performed on the raw dataset. Samples with a genotype missing rate of ≥ 0.5 and SNPs with a missing rate of ≥ 0.2 were removed. The final datasets used for testing the pipeline are shown in Table S2. Using default parameters, the pipeline successfully differentiated 1081 barley samples using 21 core SNPs, 1297 barley samples using 32 core SNPs, a merged dataset of 1081 barley samples using 19 SNPs, 800 soybean varieties using 52 SNPs, 271 wheat varieties using 24 SNPs, 453 rice accessions using 18 SNP markers, and 1206 maize varieties using 60 SNPs (Fig. 3, Table S2). The data represent the minimum set of markers obtained after running the pipeline ten times (Other results are not shown).

We compared CoreSNP with a Random Selection (RS) method using the Barley I & II dataset to evaluate their performance in selecting core SNPs. In the RS process, two markers were randomly selected from the long and short arms of each chromosome, forming a combination of 28 SNPs with aMAF greater than 0.3 (RS1) and MAF greater than 0.4 (RS2). After 20 iterations of selection, the randomly selected 28 SNPs from RS1 and RS2 identified accessions ranging from 977 to 1061. Through saturation curve analysis, we found that the CoreSNP approach exhibited significantly higher efficiency in SNP selection than the RS method, as it efficiently identified the same number of germplasms with a smaller set of selected SNPs (Fig. S1).

**Fig. 4:**
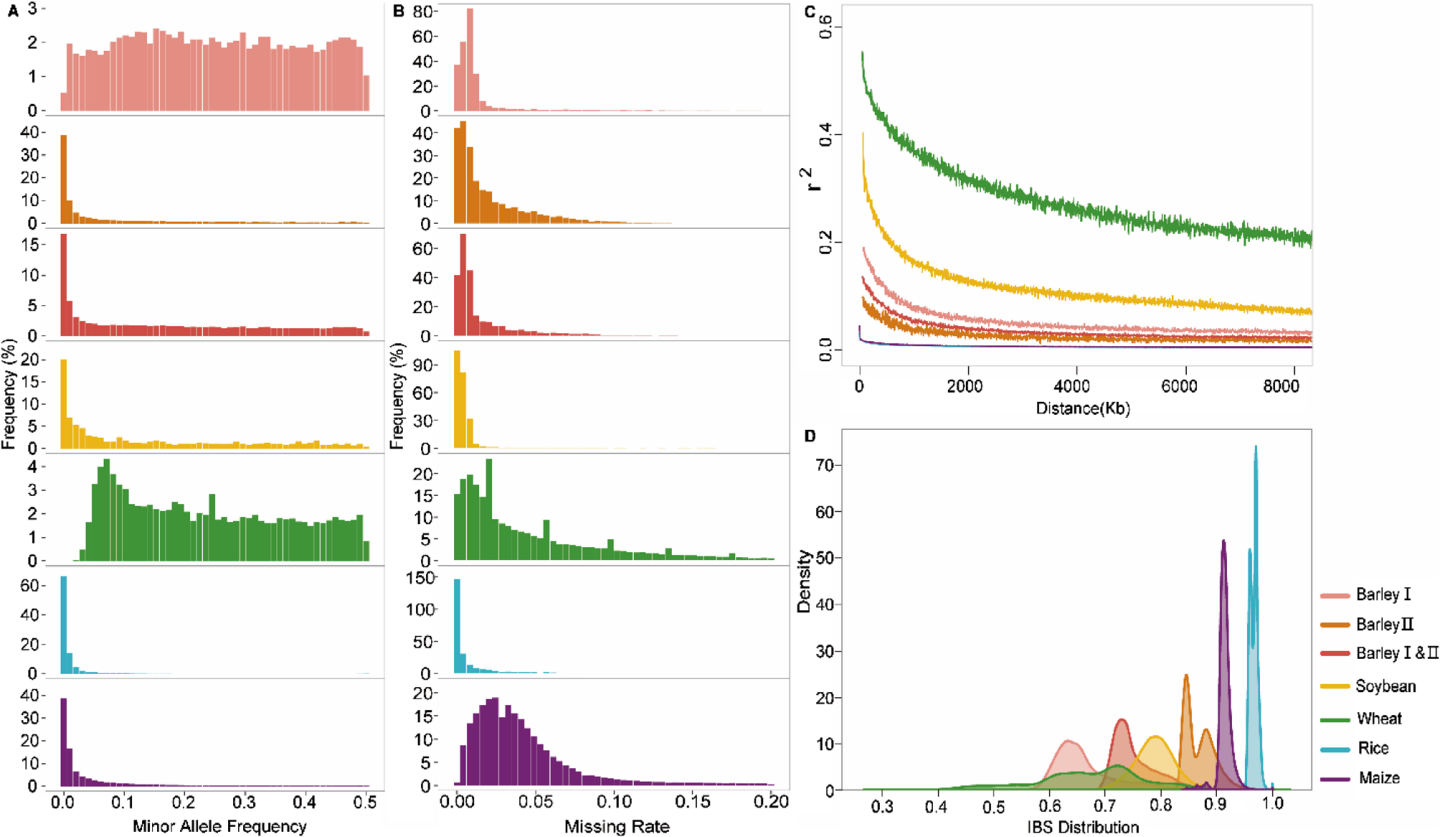
Genetic diversity of the raw test datasets. **A**, Frequency distribution of MAF values among raw dataset. **B**, Frequency distribution of PIC values among raw dataset. **C**, Linkage disequilibrium (LD) decay patterns of different species. The decays of LD (r2) with physical distance (kilobases) for SNPs in five crops are shown. **D**, Identity by states (IBS) distribution across five crops

### Characteristics of the core SNPs selected from different datasets

To assess the utility of the core SNPs, we calculated the genetic diversity parameters, including MAF and PIC, as indicators of the markers’ discriminatory ability. Values closer to 0.5 for MAF and 0.375 (depending on the formula) for PIC of biallelic markers indicate better discriminatory properties.

The results showed that more than 70% of the core SNPs had an MAF greater than 0.3 and PIC greater than 0.35, except for the soybean dataset (Fig. S2). In the case of the soybean and maize datasets, there are 6 and 8 markers, respectively, with a minor allele frequency (MAF) below 0.1. These markers were specifically chosen to distinguish individuals with high genetic similarity. Notably, all markers selected from the Barley I & II dataset exhibited MAF values greater than 0.3 and PIC values greater than 0.35, except for two markers located on chromosome 1 H and chromosome 5 H, which displayed MAF values of 0.23 and 0.19, respectively.

The 24 core markers were cover 13 chromosomes in the wheat genome, and in addition to these, the selected markers are distributed across nearly every chromosome in the genome. The majority of the core SNPs were not located in close proximity to each other, suggesting a lower LD distance between them (Supplementary Data 2).

### Genetic diversity analysis of the test datasets

Significant variations were noted in the count of the ultimate core sets during the evaluation of the CoreSNP pipeline across different datasets. To investigate the drivers underlying these disparities, we assessed several factors, encompassing MAF, missing rate, linkage disequilibrium decay distance (LDD), and identity by state (IBS) information utilizing the test dataset (Fig. 4).

No specific regularity was observed in the distribution of MAF or missing rate in soybean experiments (Fig. 4A-B). However, the soybean dataset exhibited a slower LD decay and consisted of 817 individuals, including both parental populations and their descendants (Fig. 4C). Finally, 52 markers capable of differentiating 800 samples were selected. In contrast, the maize dataset with a faster LD decay exhibited a higher missing rate and rare genetic variations. Approximately 28% of the SNPs exhibited missing rates between 0.05 and 0.2, and 20% of the SNPs had an MAF below 0.01. Consequently, during the process of selecting core SNPs in maize, we initially selected 26 markers to form the primary core, which was later expanded to include 60 SNPs after accounting for missing loci.

The wheat and barley I dataset showed a relatively uniform pattern in MAF distribution (Fig. 4A). However, the wheat dataset exhibited a higher proportion of markers with missing rates ranging from 0.05 to 0.2, slower LD decay and higher IBS peaks compared to the barley dataset (Fig. 4B-D). Consequently, the efficiency of core SNP selection was found to be lower in the wheat dataset. Under similar conditions, we were able to distinguish 1081 barley samples using 21 core markers, whereas only 271 wheat samples could be differentiated using 24 core markers (see Table S2). Overall, both the genetic diversity of the population and the specific characteristics of the sequencing data significantly influence the number of core markers.

### Flexible application of CoreSNP for barley accessions

The fertility of spikelet florets, commonly referred to as row type, and the presence or absence of grain hulls are two crucial infraspecific morphological traits in barley. In this study, we assessed the feasibility of the CoreSNP pipeline with three SNP markers (rs7_527405910 on chromosome 7 H, JHI-Hv50k-2016-107445 on chromosome 2 H, and JHI-Hv50k-2016-230985 on chromosome 4 H) that are tightly linked to these two traits as included markers for core SNP selection. By establishing a criterion of a minimum of at least two markers distinguishing each pair of samples, we successfully identified 31 core SNP markers that differentiated 1081 barley samples using the merged dataset. These core SNP markers were distributed across all seven chromosomes (Fig. S3).

The genetic structure of 1081 barley collections was analyzed using both the core SNP set and the complete set of genome-wide SNPs. Principal component analysis (PCA) revealed that some samples with relatively distant genetic distances within three clusters were still well separated along the PC1 and PC2 dimensions. This observation highlights that the core SNPs capture a certain level of the original population structure (Fig. S4).

The correlation coefficient (r) values of 0.4024 indicate a significant correlation between the genetic distance matrices of the core SNP panels and the total genome-wide SNPs. This affirms the robustness and reliability of our core SNP selection process (Table S10).

## Discussion

### Development of the CoreSNP pipeline

Appraising the discriminatory power of loci combinations is a necessary step in marker screening [50]. When assessing the performance of multiple loci combinations, MAF values have shown lower sensitivity compared to PIC values and the Shannon Diversity index, as demonstrated through statistical analysis. In the case of the frequency distribution presented in Table S11, it was observed that the Shannon index displayed a similar trend to that of the complete PIC formula. However, the computational complexity associated with calculating the full PIC formula is relatively higher than that associated with calculating the Shannon index, especially as the diversity of loci combinations expands.

In addition, we conducted a comparative analysis with the Conditional Random Selection (CRS) method using the data released in their research [34]. The results demonstrate that, for the same fractal dataset, our approach can differentiate 1000 samples with 44 SNPs, while the CRS method requires 54–59 SNPs. Additionally, due to the random sampling nature of their method, the final number of labels exhibited significant fluctuations. Furthermore, it was noted that their method has limitations in handling missing data. Thus, in the present study, we introduced CoreSNP, an screening pipeline that utilizes the Shannon index to create core sets of SNPs. Overall, the pipeline initiates from the input VCF dataset and proceeds through a fast and thorough process. CoreSNP achieved discrimination of over 1000 barley samples in just five minutes of runtime on our machine (OS: Windows 11, CPU: Intel Core i7-9850 H 2.6 GHz, RAM: 32 GB), outperforming other traditional methods [31, 32, 34].

### Polymorphic analysis of the core SNPs selected based on the different datasets

The discrimination curve of the markers was plotted based on the haplotype count obtained at each step (Fig. 2). This curve demonstrated that the SNP panels generated by this pipeline possess a remarkable discriminatory capacity. The selected core SNP markers distinguished 100% of the test collections, with the exception of 17 soybean samples and two maize samples. This discrepancy in these samples can be attributed to the MAF filtration applied to the raw dataset obtained from public sources. These particular samples represent closely related accessions, as evidenced by the calculated identical by state (IBS) information, where pairs of samples exhibited an IBS value of 1.

The selected core SNPs exhibited a high degree of polymorphism and were distributed across the genome. In fact, within the pipeline, the evaluation of an increase in haplotype numbers involved removing redundant linked markers, ensuring that adjacent pairs of SNPs were not positioned too closely to each other. The number of core SNPs is influenced by the genetic diversity of different populations. However, it is also significantly influenced by the missing rate in the original dataset. In terms of population structure, certain closely related samples are still unable to be distinctly separated in the reduced-dimensional data. This limitation could be attributed to the insufficient number of markers in comparison to the original dataset, which hindered their ability to adequately capture the genetic variations present.

### Applications of CoreSNP pipeline in the future

The cost of genome sequencing tools remains high for routine large-scale germplasm identification. However, with the development of low-density sequencing techniques, such as KASP and ARMS-PCR, our CoreSNP pipeline provides a cost-effective solution for germplasm identification and genetic relationship analysis. By constructing a reference library using a combination of core SNP alleles (haplotypes), it becomes possible to establish a comprehensive catalog of DNA profiles and DNA fingerprints for each accession. This will greatly facilitate the analysis of variety traceability and genetic backgrounds. However, it is important to note that this reference library is not a one-size-fits-all solution, thereby necessitating additional efforts in collecting more genotypes.

Moreover, these SNPs can also be utilized for parent combination selection and progeny screening during the breeding process, thus enhancing breeding efficiency and precision. Additionally, the processing of many samples inevitably introduces the issue of sample mix-up. It becomes challenging when samples contain mixtures with few identifiable characteristics. The combination of genetic profiles and core SNP alleles can be used to identify accidental sample mix-ups, ensuring the authenticity and purity of seeds. In summary, the CoreSNP pipeline takes into account sequencing platform constraints and user-specific preferences. By potentially saving time and reducing costs, it simplifies and streamlines the process of genomic identification. This tool will serve as a valuable foundation for modern breeding efforts and future germplasm management and preservation endeavors.

## Conclusion

In conclusion, we developed the CoreSNP pipeline for evolution of the discrimination power of SNP combinations. We validated it using diverse genotype files from various crops and found that it exhibited high efficiency. This tool can be efficiently used for selecting core SNPs capable of representing genome-wide variation to identify similarity and redundancy within germplasm resources.

### Electronic supplementary material

Below is the link to the electronic supplementary material.

**Supplementary Material 1**: **Fig. S1**. Comparison of the CoreSNP pipeline and Random Selection(RS) method for SNP selection. **Fig. S2**. Frequency distribution of MAF and PIC values among the selected core SNPs. **Fig. S3**. Distribution of core SNPs selected from barley merged dataset with specific parameters. **Fig. S4**. The comparison of the Principle Component Analysis (PCA) analysis based on the raw genome-wide SNPs and core SNPs. **Table S1**. Multiple options for running the core SNP pipeline. **Table S2**. Datasets description and core SNP selection in various crop species. **Table S10**. Mantel’s test for comparisons among genetic distance matrices calculated using the core SNPs and the original dataset. **Table S11**. Comparison of MAF, PIC and Shannon index among different haplotypes

**Supplementary Material 2**: **Table S3**. Description of Core SNPs Selected from the BarleyIdataset with default parameters. **Table S4**. Description of Core SNPs Selected from the BarleyIdataset with default parameters. **Table S5**. Description of Core SNPs Selected from the BarleyI&IIdataset with default parameters. **Table S6**. Description of Core SNPs Selected from the Soybean dataset with default parameters. **Table S7**. Description of Core SNPs Selected from the Wheat dataset with default parameters. **Table S8**. Description of Core SNPs Selected from the Rice dataset with default parameters. **Table S9**. Description of Core SNPs Selected from the Maize dataset with default parameters

## Data Availability

The original contributions presented in the study are included in the article and Supplementary materials.
