# Supplementary material for: CoreSNP: an efficient pipeline for core marker profile selection from genome-wide SNP datasets in crops
Source: BMC Plant Biol. 2023 Nov 21;23:580. doi: 10.1186/s12870-023-04609-w (PMC10662547; doi:10.1186/s12870-023-04609-w)
Supplement: Supplementary file 1 — Supplementary Material 1: Fig. S1. Comparison of the CoreSNP pipeline and Random Selection(RS) method for SNP selection. Fig. S2. Frequency distribution of MAF and PIC values among the selected core SNPs. Fig. S3. Distribution of core SNPs selected from barley merged dataset with specific parameters. Fig. S4. The comparison of the Principle Component Analysis (PCA) analysis based on the raw genome-wide SNPs and core SNPs. Table S1. Multiple options for running the core SNP pipeline. Table S2. Datasets description and core SNP selection in various crop species. Table S10. Mantel’s test for comparisons among genetic distance matrices calculated using the core SNPs and the original dataset. Table S11. Comparison of MAF, PIC and Shannon index among different haplotypes [file 12870_2023_4609_MOESM1_ESM.docx]

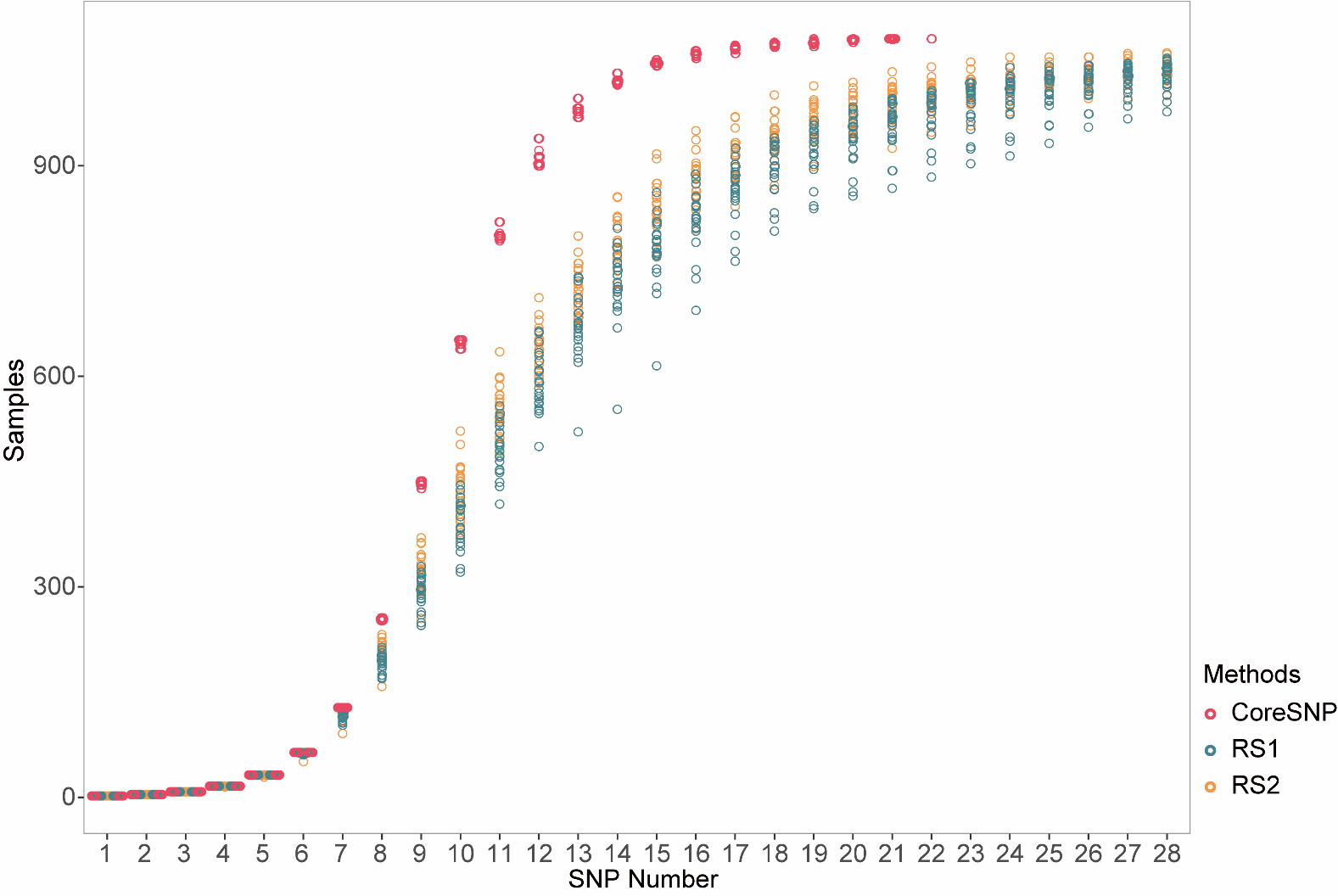


Fig. S1. Comparison of the CoreSNP pipeline and Random Selection(RS) method for SNP selection. RS1, random selection of 28 SNPs from the dataset with a MAF greater than 0.3. RS2, random selection of 28 SNPs from the dataset with MAF greater than 0.4.


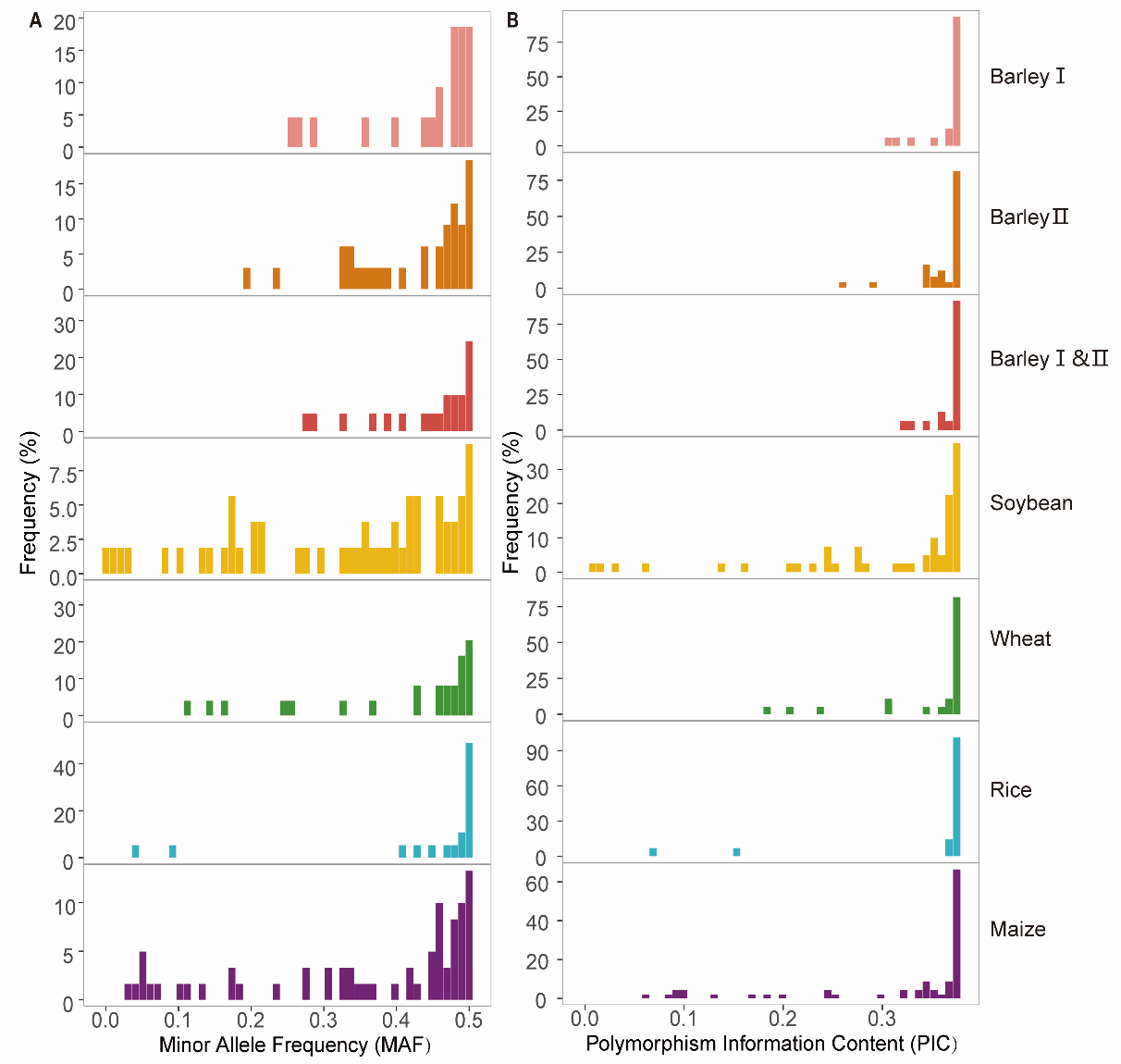


Fig. S2. Frequency distribution of MAF and PIC values among the selected core SNPs. A, MAF values; B, PIC values.


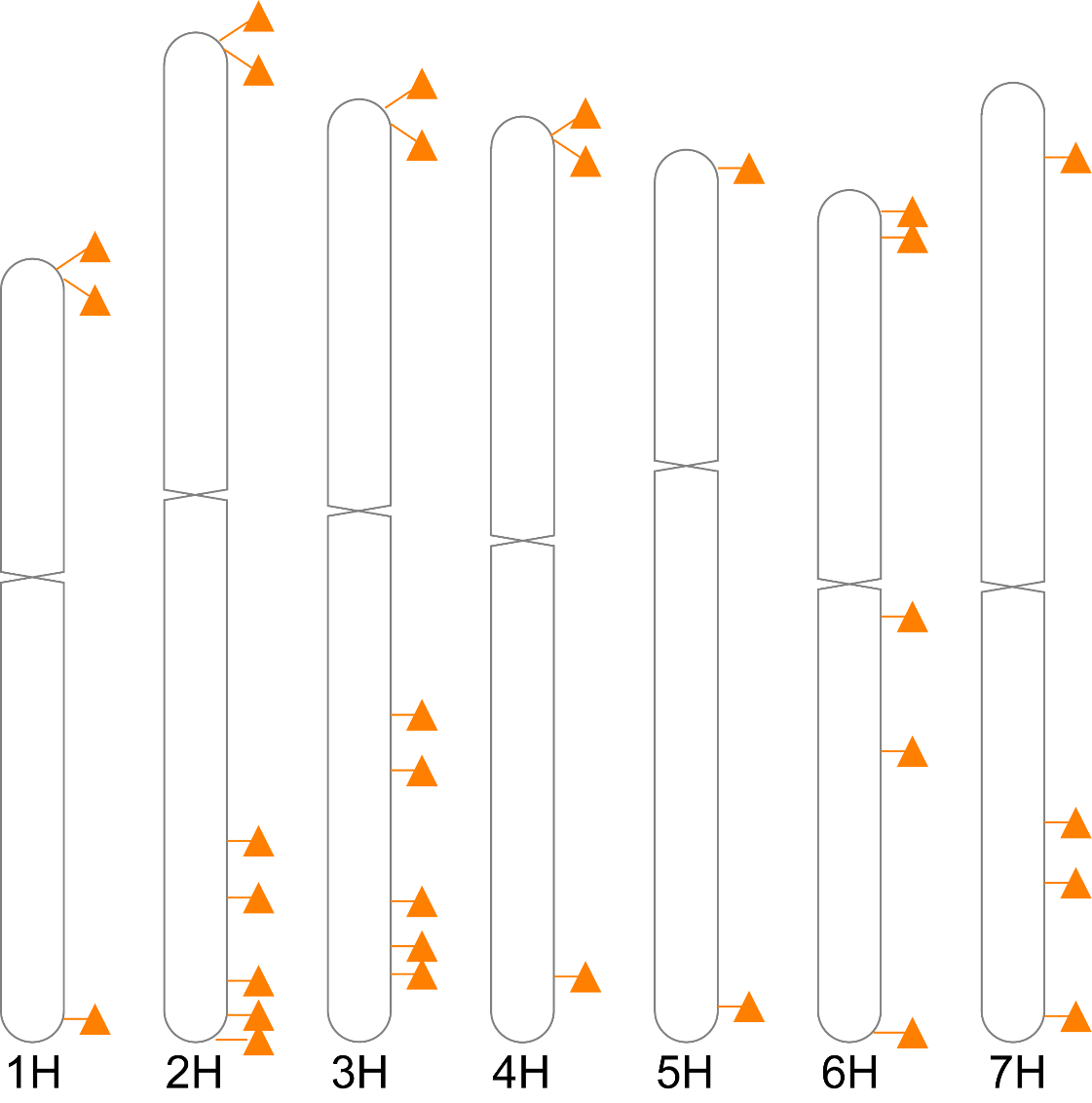


Fig. S3. Distribution of core SNPs selected from barley merged dataset with specific parameters.


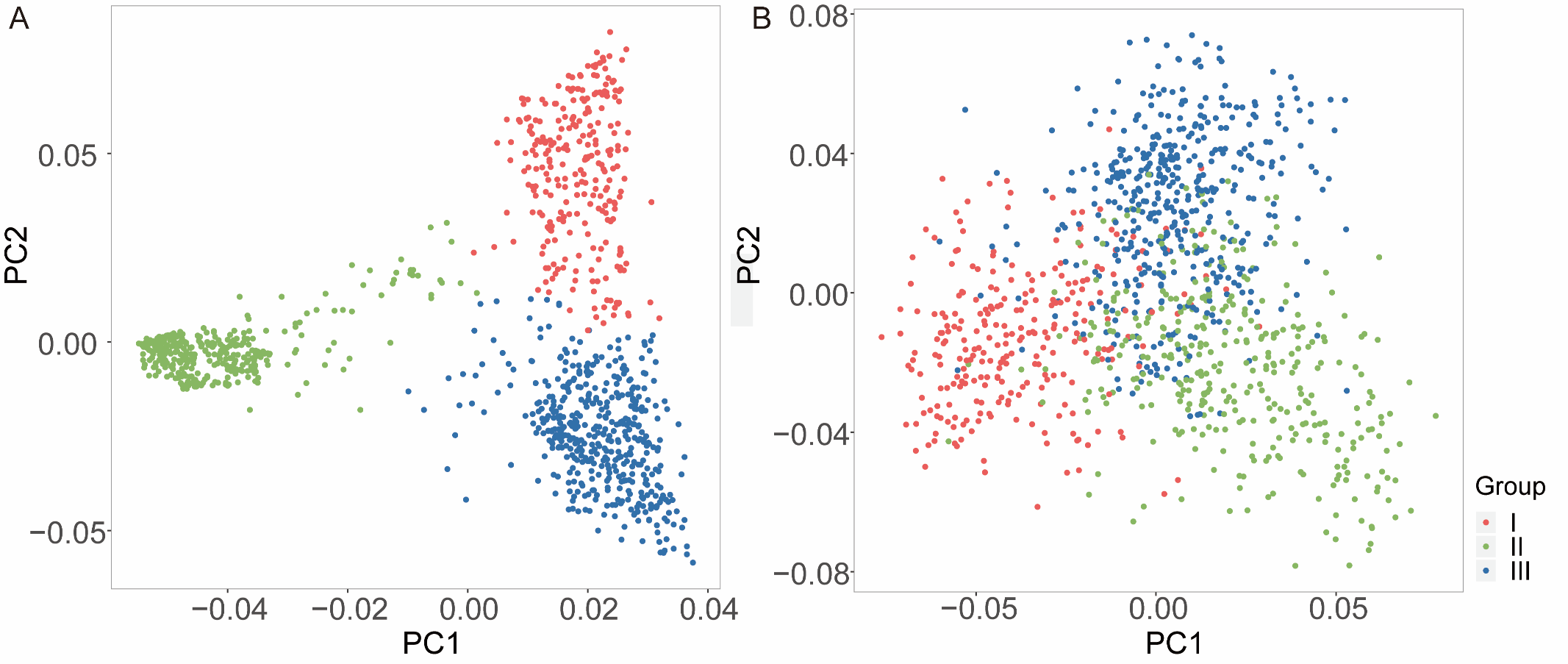


Fig. S4. The comparison of the Principle Component Analysis (PCA) analysis based on the raw genome-wide SNPs and core SNPs. A, the PCA analysis is conducted using the entire set of genome-wide SNPs. B, the PCA analysis is conducted using the core SNPs selected by the CoreSNP pipeline.

Table S1. Multiple options for running the core SNP pipeline

| Options | Type | Description and Notes |
| --- | --- | --- |
| -v/--vcf | <str> | Input VCF file, gzip compressed file is supported. |
| -i/--include | <str> | A file contained SNPs that must be included in the core set. |
| -e/--exclude | <str> | A file contained SNPs that would never be included in the core set. |
| -x/--flexing | <int> | The minimal number of candidate SNPs at each round. (default: 1, range: 1~5) |
| -m/--minimal | <int> | The minimal number of differential SNPs for each pairwise samples. (default: 1, range: 1~2) |
| -c/--count | <int> | The number of core sets this program generates. (default: 1, range: 1~10) |
| -g/--missing | <float> | The threshold of missing call frequency to filter variants. (default: 0.2, range: 0~0.5) |
| -f/--maf | <float> | The threshold of minor allele frequency to filter variants. (default: 0, range: 0~0.5) |
| -o/--out | <str> | Directory name of output results. (default: result) |
| -l/--log | <str> | Filename of log file. (default: coreSNP.log) |
| -M/--more-info | <str> | Print more info to log when running. |

Table S2. Datasets description and core SNP selection in various crop species.

| Crops | Genotyping Platforms | Samples | Number of SNPs | Number of SNPs with Imputation and Filtration | No. of Final Core SNPs | Reference |
| --- | --- | --- | --- | --- | --- | --- |
| BarleyⅠ | Illumina 50K Array | 1,081 | 42,520 | 40,019 | 21 | Darrier B, et al. 2019 |
| BarleyⅡ | GBS | 1,297 | 143,413 | 34,127 | 32 | Milner SG, et al. 2019 |
| BarleyⅠ&Ⅱ | Merged dataset | 1,081 | 185,508 | 73,241 | 19 |  |
| Soybean | Illumina Array | 817 | 106,197 | 96,889 | 52 | Sun R, et al. 2022 |
| Wheat | Axiom 660K Array | 271 | 178,803 | 165,905 | 24 | Yao F, et al. 2021 |
| Rice | WGS | 453 | 18,128,777 | 1,166,242 | 18 | Wang W, et al. 2018 |
| Maize | WGS | 1,210 | 1,661,576 | 575,480 | 57 | Bukowski R, et al. 2018 |

GBS, Genotyping by sequencing; WGS, Whole genome sequencing

Table S10. Mantel's test for comparisons among genetic distance matrices calculated using the core SNPs and the original dataset.

| Core Sets | Counts | Mantel's | | |
| --- | --- | --- | --- | --- |
|  |  | r | z-score | p |
| Core_1 | 31 | 0.3781 | 82.1972 | 0.0001 |
| Core_2 | 31 | 0.3244 | 72.5266 | 0.0001 |
| Core_3 | 31 | 0.4024 | 84.6475 | 0.0001 |
| Core_4 | 32 | 0.3244 | 72.6945 | 0.0001 |
| Core_5 | 31 | 0.3439 | 78.7137 | 0.0001 |
| Core_6 | 31 | 0.3781 | 82.4422 | 0.0001 |
| Core_7 | 31 | 0.3244 | 72.5391 | 0.0001 |
| Core_8 | 31 | 0.4024 | 84.7437 | 0.0001 |
| Core_9 | 32 | 0.3244 | 72.7753 | 0.0001 |
| Core_10 | 31 | 0.3440 | 78.9793 | 0.0001 |

Table S11. Comparison of MAF, PIC and Shannon index among different haplotypes.

|  | S1 | S2 | S3 | S4 | S5 | S6 | S7 | S8 | S9 | S10 | S11 | S12 | Frequency | MAF | PIC (Simplified) | PIC (Fulled) | Shannon Index |
| --- | --- | --- | --- | --- | --- | --- | --- | --- | --- | --- | --- | --- | --- | --- | --- | --- | --- |
| M1M2M3 | AAA | AAA | AAA | AAA | AGC | AGC | AGC | AGC | TAC | TAC | TAC | TAC | 4,4,4 | 0.3333 | 0.6667 | 0.5926 | 1.0986 |
| M1M2M4 | AAA | AAA | AAA | AAA | AAA | AGC | AGC | AGC | AGC | TAC | TAC | TAC | 5,4,3 | 0.3333 | 0.6528 | 0.5786 | 1.0776 |
| M1M2M5 | AAA | AAA | AAA | AGC | AGC | AGC | TAC | TAC | TAC | TAC | TAC | TAC | 3,3,6 | 0.2500 | 0.6250 | 0.5547 | 1.0397 |
| M1M2M6 | AAA | AAA | AAA | AAA | AAA | AGC | AGC | AGC | AGC | AGC | TAC | TAC | 5,5,2 | 0.1677 | 0.6250 | 0.5454 | 1.0282 |

AAA, AGC, TAC, different haplotypes of six markers (M1-M6) in twelve samples (S1-S12). It is assumed that there are only three combinations in twelve samples and markers M4-M6 exhibit the same allele pattern across all samples.
